# Case report: Osteosarcomatous differentiation in the lung metastasis of a malignant phyllodes tumor

**DOI:** 10.3389/fmed.2023.1141353

**Published:** 2023-03-21

**Authors:** Ruijing Liu, Jingli Xue, Wen Liu, Beibei Jiang, Fuyun Shi, Zhenzheng Wang, Peifeng Li

**Affiliations:** ^1^Department of Pathology, The Postgraduate Training Base of Jinzhou Medical University (The 960th Hospital of PLA), Jinan, China; ^2^Department of Pathology, The 960th Hospital of PLA, Jinan, China; ^3^Department of Pathology, The Fourth People’s Hospital of Jinan, Jinan, China

**Keywords:** malignant phyllodes tumor, osteosarcomatous differentiation, liposarcomatous differentiation, tumor metastasis, cancer stem cells, case report

## Abstract

Malignant phyllodes tumor is a rare breast tumor, with distant metastases and heterologous differentiation in a few cases. We report a case of malignant phyllodes tumor with liposarcomatous differentiation in the primary tumor and osteosarcomatous differentiation in the lung metastatic tumor. A middle-aged female presented with a well-defined mass in the upper lobe of the right lung measuring 5.0 × 5.0 × 3.0 cm. The patient had a history of malignant phyllodes tumor in the breast. The patient underwent a right superior lobectomy. Histologically, the primary tumor was a typical malignant phyllodes tumor with pleomorphic liposarcomatous differentiation, while the lung metastasis showed osteosarcomatous differentiation without original biphasic features. The phyllodes tumor and heterologous components showed CD10 and p53 expression, and were negative for ER, PR, and CD34. Exome sequencing revealed *TP53*, *TERT*, *EGFR*, *RARA*, *RB1*, and *GNAS* mutations in all three components. Although the lung metastasis were morphologically different from the primary breast tumor, their common origin was demonstrated through immunohistochemical and molecular characterization. Cancer stem cells give rise to tumor heterogeneous cells, and heterologous components in malignant phyllodes tumors may indicate unfavorable prognosis and a greater risk of early recurrence and metastasis.

## Introduction

Phyllodes tumor is an uncommon, fibroepithelial neoplasm with leaf-like stromal fronds that can be classified as benign, borderline, or malignant based on the histological characteristics. Malignant phyllodes tumors (MPTs) commonly occur between the ages of 45 and 49 years (1). Marked stromal nuclear pleomorphism and stromal overgrowth, increased mitoses, and infiltrative borders can usually be seen. Some tumors may show heterologous differentiation, including liposarcomatous, fibrosarcomatous, osteosarcomatous, chondrosarcomatous, and rhabdomyosarcomatous structures, indicating a poorer prognosis (2–4). Distant metastases occur in 23% of MPTs and show histological features similar to the primary tumor (5). We report a case of pleomorphic liposarcomatous primary MPT with osteosarcomatous lung metastasis.

## Case presentation

**Figure 1:**
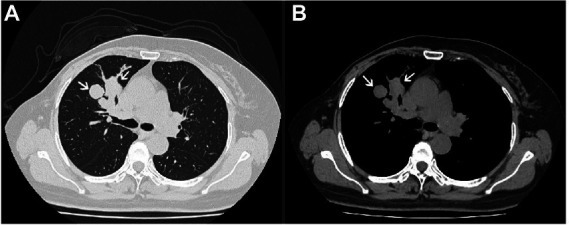
Chest computed tomography showing a soft-tissue density mass in the upper lobe of the right lung. **(A)** lung window; **(B)** mediastinal window.

A 57-year-old female was admitted with a right lung mass detected on physical examination 3 days previously. Chest computed tomography revealed a soft-tissue density mass in the upper lobe of the right lung near the hilum, with bronchial obstruction in the anterior segment of the upper lobe and a patchy fuzzy shadow distally (Figure 1). Laboratory findings, including tumor markers, were within the normal ranges. The patient had a history of MPT of the breast with liposarcomatous differentiation removed 1.5 years previously. Then, a right superior lobectomy was performed. No adjuvant treatments were administered after lung resection, and the patient underwent an uneventful recovery without tumor recurrence during 1 year of follow-up. This study was approved by the 960th Hospital’s institutional ethical committee, and performed in accordance with the principles embodied in the Declaration of Helsinki.

**Figure 2:**
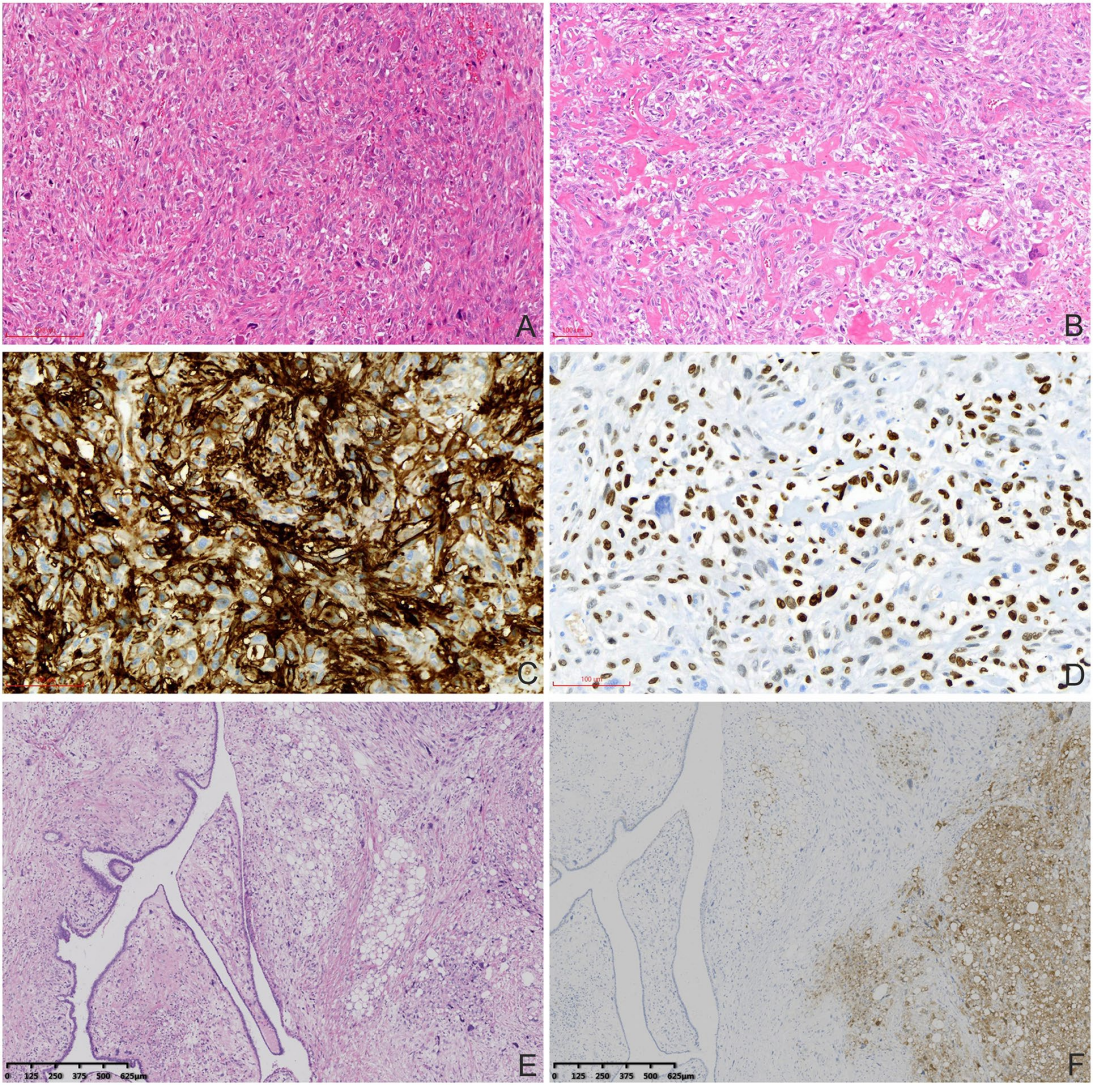
Histopathological and immunohistochemical characteristics of the MPTs. **(A)** Atypical elongated cells had considerable diversity in the lung metastatic tumors (haematoxylin and eosin staining, 100×). **(B)** Abundant osteoclasts and osteoid formation were observed (haematoxylin and eosin staining, 100×). Tumor cells were positive for CD10 **(C)** and SATB-2 (**D**; immunohistochemical staining, 200×). **(E)** The MPT (left) and pleomorphic liposarcomatous differentiation (right) were mixed without clear boundaries in the primary breast MPT (haematoxylin and eosin staining, 40×). **(F)** Tumor cells with pleomorphic liposarcomatous differentiation were positive for S-100 (right; immunohistochemical staining, 40×).

Macroscopic examination showed a multinodular, solid, grey-white, medium-textured mass measuring 5.0 × 5.0 × 3.0 cm. Histopathologically, the tumor was composed of spindle, ovoid, and osteoclast-like giant cells with focal osteoid formation. Atypical elongated cells had considerable morphological diversity with enlarged, hyperchromatic, and lobulated nuclei. The tumor cells were arranged in sheets or intertwined fascicles. There was multifocal abundance of osteoclast-like multi-nucleated giant cells. Some portions of the mesenchyme contained areas of osteoid formation (Figures 2A,B). Local necrosis and cystic changes were also noted. The mitotic index was 12 mitoses per 10 high-power fields (HPF). Irregular tumor nodules bulged into the surrounding lung tissue. Hilar nodes were free of tumor cells. The immunohistochemistry analysis is shown in Table 1 and Figures 2C,D. The lung tumor was pathologically diagnosed as metastatic breast MPT with osteosarcomatous differentiation. The lung tumor was histologically different from the original breast tumor, with significant cell density and atypia.

**Table 1:** Immunohistochemical characteristics of different components of the malignant phyllodes tumor.

Antibody	Malignant phyllodes tumor	Liposarcomatous differentiation	Osteosarcomatous differentiation
CD10	+	+	+
P53	+	+	+
ER	−	−	−
PR	−	−	−
CD34	−	−	−
CD117	local +	−	−
SMA	local +	−	local +
Actin	local +	−	local +
S-100	−	+	−
SATB-2	−	−	+
Ki-67	25%	35%	30%

The pathological features of the primary breast tumor represented a mixture of phyllodes tumor and liposarcoma (Figures 2E,F). Mesenchymal lesions had caused marked narrowing of the tubular epithelial structures. There was some mesenchymal cell atypia with large, irregular, hyperchromatic nuclei. Pleomorphic liposarcomatous components were observed. Eight mitoses per 10 HPF and 2 per 10 HPF were observed in areas of phyllodes tumor and liposarcomatous differentiation, respectively. The tumor had poorly defined borders but there was no obvious axillary lymph node enlargement.

Three different morphological components of MPT and normal breast tissue were detected using GENESEEQ PRIME™ high-throughput DNA sequencing (Geneseeq Technology Inc., Nanjing, China), which targets the coding regions of 425 cancer-related genes. All three components showed *TP53*, *TERT*, *EGFR*, *RARA*, *RB1*, and *GNAS* gene mutations with similar frequencies (Table 2). *MET* and *PREX2* missense mutations were detected only in the liposarcomatous component and lung metastasis, respectively, with low mutation frequency.

**Table 2:** Mutation frequencies of different components in the malignant phyllodes tumor.

Gene	Alteration	ExonicFunc	Malignant phyllodes tumor	Liposarcomatous differentiation	Osteosarcomatous differentiation
*TERT*	c.-124C > T	upstream_gene_variant	34.97%	36.36%	40.71%
*EGFR*	c.3114 + 1G > T	splice_donor_variant	35.71%	32.35%	33.55%
*TP53*	c.832C > A	missense_variant	67.44%	60.87%	58.87%
*RARA*	c.701_703delAGT	inframe_deletion	57.58%	78.26%	58.04%
*RB1*	c.1421G > C	missense_variant	75.00%	55.56%	47.01%
*GNAS*	c.1727C > A	missense_variant	22.14%	35.42%	54.52%
*MET*	c.901A > C	missense_variant	-	25.00%	-
*PREX2*	c.2570C > A	missense_variant	-	-	15.61%

## Discussion

Breast MPTs are rare tumors that account for <1% of all breast tumors. Recurrences occur in 23–30% of the cases (6, 7), but few tumors metastasize to other parts of the body, including lungs, bones, ovaries, brain, and pancreas, mainly through the hematogenous route (8–10). Our patient underwent a mastectomy for breast MPT, but the tumor metastasized to the lung 1.5 years after the surgery, indicating the aggressive nature of the MPT. Surgical excision with clear margins without lymphadenectomy remains the cornerstone of treatment for localized MPTs. The use of chemotherapy remains controversial (11). The treatment of metastatic tumors involves chemotherapy using conventional drugs for the sarcomatoid component. The patient chose a conservative treatment strategy, and no adjuvant therapy was performed after pulmonary lobectomy. No tumor recurrence was observed during 1 year follow-up.

Cancer stem cells give rise to tumor heterogeneous cells and form heterogeneous components. Liposarcomatous differentiation is common in heterogeneous MPTs (4). Unlike well-differentiated liposarcomas, pleomorphic liposarcomas increase the metastatic risk of breast phyllodes tumors. In the present case, breast MPT with pleomorphic liposarcomatous differentiation metastasized 1.5 years after mastectomy with negative margins, indicating the influence on the malignant potential. Osteosarcomatous differentiation of MPTs is rare, accounting for 1.3% of breast phyllodes tumors (12). The osteosarcomatous component comprises a variable percentage of MPTs, ranging from 25 to 100%. Osteosarcomatous differentiation in primary or recurrent tumors indicates a poor prognosis. Silver et al. (12) investigated 22 cases of osteosarcomatous MPTs and found that their biological behavior was similar to that of soft tissue osteosarcomas, with distant metastases and tumor-related deaths occurring in 38 and 33% of the patients, respectively. Osteosarcomatous differentiation only in the metastatic tumors is rare in breast MPTs. The definitive diagnosis was based on the histopathology, immunochemistry, and the molecular characteristics. Tsubochi et al. (13) reported a similar case with osteosarcomatous component detected only in the lung metastasis; the patient remained disease-free 2 years after pulmonary lobectomy. Despite liposarcomatous differentiation of the primary breast tumor in this patient, there was no tumor recurrence during the 1-year follow-up after lung tumor resection. Osteosarcomatous breast MPTs have been reported to be biologically aggressive tumors characterized by early recurrence and hematogenous metastasis, frequently to the lungs (14). However, the prognostic value of osteosarcomatoid differentiation in MPT metastases needs to be confirmed in further studies.

In this case, only osteosarcomatoid component was found in the metastatic lung tumor; no MPT or lipomarcomatoid components were observed. It is important to distinguish these tumors from other neoplasms that may contain osseous structures, such as primary extraosseous osteosarcomas, carcinosarcomas, and ossifying stromal tumors. Immunohistochemical and molecular genetic analyses may be used for differential diagnosis. In the present case, the three components were positive for CD10 and p53 on immunohistochemical staining and had similar molecular mutation characteristics. To the best of our knowledge, the genetic characteristics of different heterologous components of MPTs have not been previously compared used high-throughput sequencing.

In conclusion, breast MPTs may develop heterologous differentiation in the primary or metastatic tumors, which indicates an unfavorable prognosis. This was a unique case with pleomorphic liposarcomatous differentiation in the primary tumor and osteosarcomatous differentiation in the lung metastatic tumor. The heterologous components showed immunohistochemical and molecular characteristics similar to MPTs. Careful follow-up is essential in MPT patients with heterologous differentiation.

## Data availability statement

The original contributions presented in the study are included in the article/supplementary material, further inquiries can be directed to the corresponding author.

## Ethics statement

The studies involving human participants were reviewed and approved by the 960th Hospital’s institutional ethical committee. The patients/participants provided their written informed consent to participate in this study. Written informed consent was obtained from the individual(s) for the publication of any potentially identifiable images or data included in this article. Written informed consent was obtained from the participant/patient(s) for the publication of this case report.

## Author contributions

RL: conceptualization, data curation, and writing – original draft. JX, WL, BJ, FS, and ZW: data curation and investigation. PL: conceptualization, project administration, and writing – review and editing. All authors contributed to the article and approved the submitted version.

## Conflict of interest

The authors declare that the research was conducted in the absence of any commercial or financial relationships that could be construed as a potential conflict of interest.

## Publisher’s note

All claims expressed in this article are solely those of the authors and do not necessarily represent those of their affiliated organizations, or those of the publisher, the editors and the reviewers. Any product that may be evaluated in this article, or claim that may be made by its manufacturer, is not guaranteed or endorsed by the publisher.

## Abbreviations

MPTs: malignant phyllodes tumors
HPF: high power fields
